# Development and evaluation of Goal setting and Action Planning (G-AP) training to support person-centred rehabilitation practice

**DOI:** 10.3389/fresc.2025.1505188

**Published:** 2025-03-31

**Authors:** Lesley Scobbie, Katie Elliott, Sally Boa, Lynn Grayson, Emily Chesnet, Iona Izat, Mark Barber, Rebecca Fisher

**Affiliations:** ^1^ School of Health and Life Sciences, Glasgow Caledonian University, Glasgow, United Kingdom; ^2^ Stroke MCN Team, NHS Lanarkshire, Coatbridge, United Kingdom; ^3^ Education, Research and Practice Development, Strathcarron Hospice, Denny, United Kingdom; ^4^ Brain Injury Rehabilitation Service, NHS Lanarkshire, Hamilton, United Kingdom; ^5^ North Stroke and Neurological Rehabilitation Team, NHS Lanarkshire, Coatbridge, United Kingdom; ^6^ University Hospital Monklands, NHS Lanarkshire, Airdrie, United Kingdom; ^7^ Stroke Programme, King’s College London, London, United Kingdom

**Keywords:** training, rehabilitation, goal setting, person-centred, implementation, evaluation, mixed methods

## Abstract

**Background:**

Stroke survivor's goals reflect their individual priorities and hopes for the future. Person-centred goal setting is recommended in rehabilitation clinical guidelines, but evidence-based training to support its implementation in practice is limited. We aimed to develop, describe and evaluate a new Goal setting and Action Planning (G-AP) rehabilitation training resource to support person-centred goal setting practice in community neuro-rehabilitation settings.

**Methods:**

A clinical-academic team, advisory group and web-design company were convened to co-develop the G-AP training resource. G-AP training was then delivered to multi-disciplinary staff (*n* = 48) in four community neuro-rehabilitation teams. A mixed methods evaluation utilising a staff questionnaire and focus group discussion was conducted to investigate staff experiences of G-AP training and their early G-AP implementation efforts. Questionnaire data were analysed descriptively; focus group data were analysed using a Framework approach. An integrated conceptual overview of data was developed to illustrate findings.

**Results:**

A fully online G-AP training resource comprising a training website and two interactive webinars was developed. Following training, 41/48 (85%) staff completed the online questionnaire and 8/48 (17%) participated in the focus group. Nearly all staff rated the training website as excellent (*n* = 25/40; 62%) or good (*n* = 14/40; 35%) and the webinars as excellent (*n* = 26/41; 63%) or good (*n* = 14/41; 34%). Following training, staff agreed they were knowledgeable about G-AP (37/41; 90%) and had the confidence (35/40; 88%) and skills (35/40; 88%) to use it in practice. Within one month of training, staff described implementing G-AP individually, but transitioning to implementation at a team level required more time to develop new working practices. Team context including staff beliefs about G-AP, leadership support and competing demands impacted (positively and negatively) on staff training engagement, learning experience and implementation efforts.

**Conclusions:**

The new G-AP training resource was positively evaluated and supported early G-AP implementation efforts. This study advances our understanding of training evaluation by highlighting the training—context interaction the temporal nature of training effects. A follow up study evaluating longer term G-AP implementation is underway.

## 1 Introduction

Person-centred goal setting is a cornerstone of good neurological rehabilitation practice (1) and is recommended in rehabilitation policy (2, 3) and clinical guidelines (4, 5). Working in partnership with patients who have neurological conditions to identify and pursue their personal goals optimises their motivation and involvement in the goal setting process (6, 7) and ensures that rehabilitation addresses their needs, preferences and priorities (8–10). However, evidence suggests that routine goal setting practice is sub-optimal (11–13) and that rehabilitation staff would like targeted training to support their goal setting practice (13, 14).

Evidence and theory based (15–17), the Goal setting and Action Planning (G-AP) framework informs a person-centred approach to the setting and pursuit of rehabilitation goals. G-AP is delivered by multidisciplinary community rehabilitation teams and its implementation tailored to individual patients within local contexts. A G-AP record provides patients with a copy of their personal goals, plans and progress (17); an accessible version (Access G-AP) is available for patients with communication difficulties (18).

A G-AP training prototype including both online and face-to-face components has been developed to support person-centred goal-setting practice (19). To enhance G-AP-related knowledge, skills, confidence, and practice, behaviour change techniques including role play, information provision, feedback, and modelling are incorporated (20, 21). The training has been positively evaluated by rehabilitation staff (19); however, additional training content was recommended to support planning for G-AP implementation in local contexts and provision of accessible resources to support patients with communication difficulties throughout the G-AP process. Additionally, the onset of COVID-19 required a fully online training resource to comply with social distancing restrictions.

Whilst staff training is the primary strategy used to support implementation of new rehabilitation interventions (22), training development and evaluation is consistently under reported (23). We sought to address this evidence-practice gap by developing, describing and conducting an initial evaluation of a new online G-AP training resource (based on the protype) to support delivery of person-centred goal setting practice in community neuro-rehabilitation settings. The research questions we sought to answer were:

**RQ1** What are staff opinions and experiences of the new online G-AP training resource?
**RQ2** To what extent does the new online G-AP training resource prepare staff to deliver G-AP in practice?

## 2 Methods

### 2.1 Phase 1: development of the online G-AP training resource

#### 2.1.1 Co-production approach

A clinical-academic project team (*n* = 5) and advisory group (*n* = 8) were convened to develop the new online G-AP training resource. The clinical-academic team comprised of five practicing neuro-rehabilitation clinicians including three speech and language therapists (SB, EC, LG), one occupational therapist (LS) and one physiotherapist (II). In addition to their clinical role, LS and SB were academics with expertise in G-AP research. The advisory group comprised of two carers and three people with neurological conditions, including one with a communication difficulty. Three rehabilitation staff members completed the advisory group (See Supplementary File S1). A web design company with video production and graphic design expertise worked alongside the project team to create the new G-AP training website.

#### 2.1.2 Development of new training content

Research evidence, clinical experience, rehabilitation policy and advisory group feedback informed updates and additions to the online G-AP training prototype, including new content and resources to support local G-AP implementation and delivery of G-AP to patients with communication difficulties (see Supplementary File S2). Webinars were introduced to replace the face-to-face training but retain the interactive and peer learning component of the training.

#### 2.1.3 Use of implementation strategies

**Figure 1:**
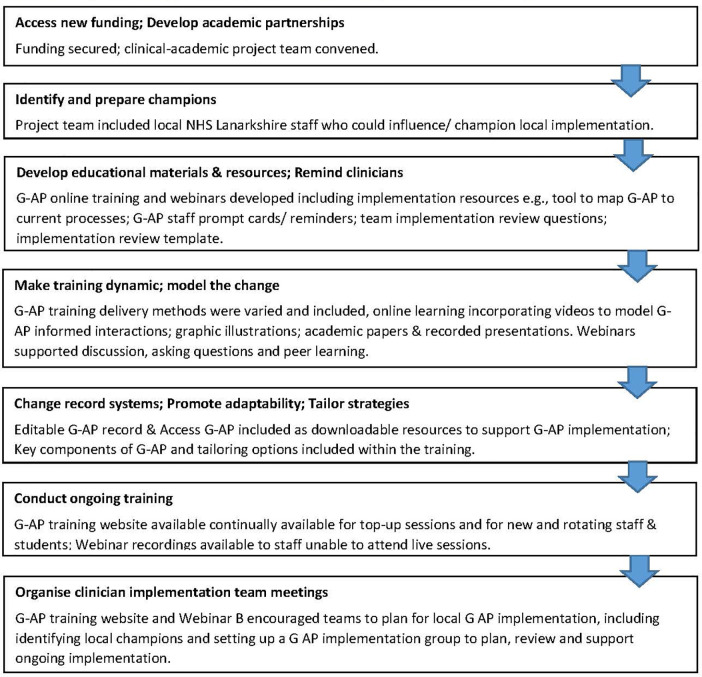
Strategies used to support G-AP implementation (ERIC compilation; Powell et al., 2015).

Implementation strategies defined as “methods or techniques used to improve adoption, implementation, sustainment, and scale-up of interventions” (24) informed the project set up and the development and delivery of the training materials and resources. The specific implementation strategies used, described using the Expert Recommendations for Implementing Change (ERIC) compilation (25), are summarised in Figure 1.

A content overview of the developed online G-AP training resource, including the training website and interactive webinars is presented in Table 1.

**Table 1:** Overview of the developed online G-AP training resource.

**G-AP training website: freely available: https://g-apframework.scot/**
○ **Home Page:** Welcome to the G-AP training; training objectives ○ **About G-AP:** Why use G-AP? G-AP theory and evidence; Positivity and Hope ○ **G-AP Training:** Descriptor of each G-AP stage (what, how, why, outcome); Role-play videos illustrating delivery of each G-AP stage in practice; examples of how to write Goals, Plans and Progress in the G-AP record. ○ **Rights, Barriers and Ramps:** Information, video clips and strategies about supporting people with cognitive and or communication difficulties through the G-AP process. ○ **Implementation:** Information and resources to help teams plan for local G-AP implementation. ○ **Resources:** Downloadable versions of the person held G-AP and Access G-AP record, relevant academic papers and recorded conference presentations.
**Interactive G-AP webinars: 2 × 2-hour interactive sessions delivered on MS teams**
○ **Webinar A:** Case study based discussion to support shared learning about supporting people with neurological problems through the G-AP process. In particular, it focusses on common ‘clinical dilemmas’ and how to manage them in practice. ○ **Webinar B:** Supports teams to think about local G-AP implementation. Evidence based implementation strategies are introduced and local implementation plans discussed.

### 2.2 Phase 2: delivery and evaluation of the online G-AP training resource

**Figure 2:**
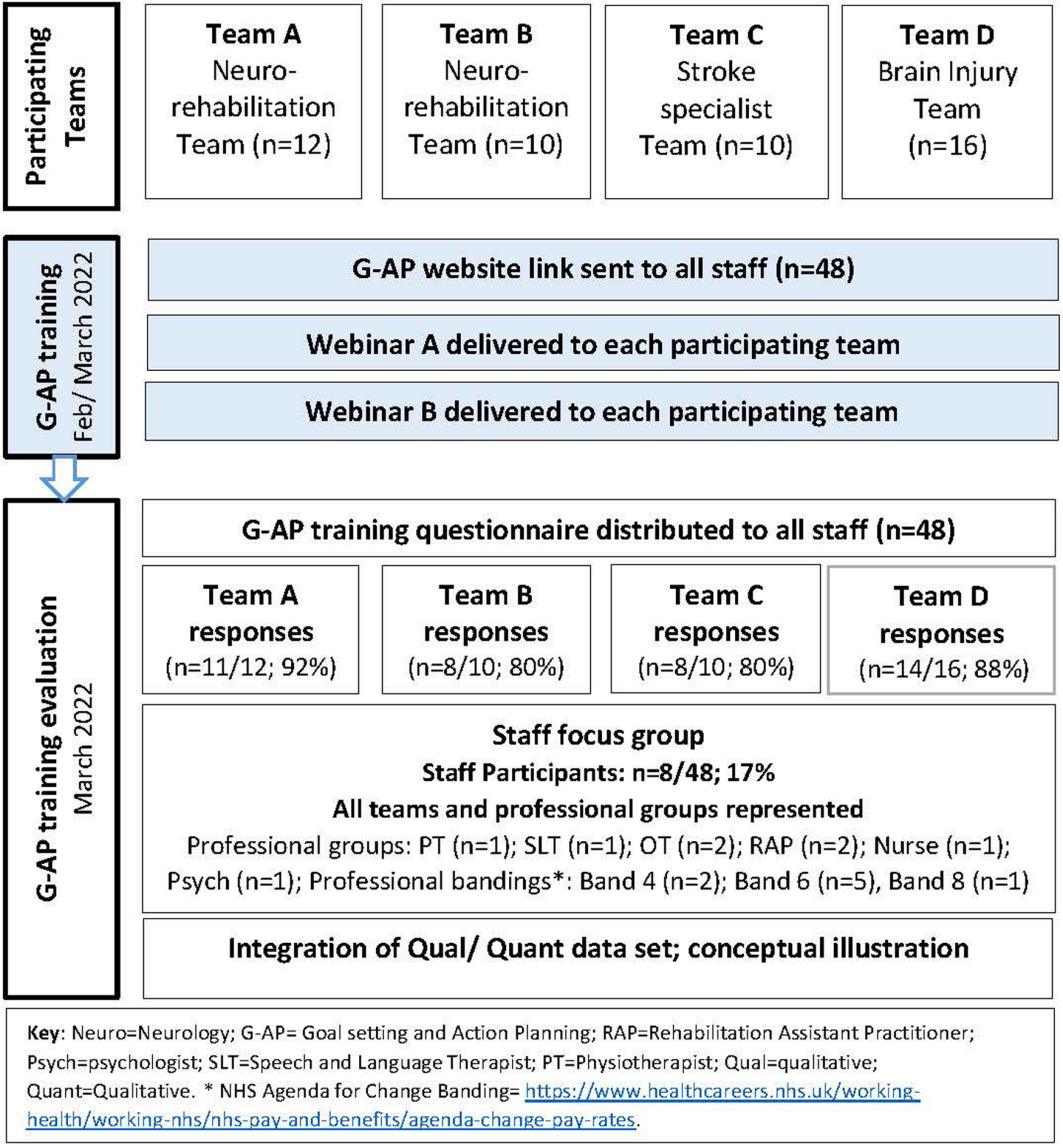
Study participants and procedure.

An overview of the study participants and procedure is summarised in Figure 2.

#### 2.2.1 Participating community teams

Two neuro-rehabilitation teams (Team A and B) supported by a specialist stroke team (Team C) and one brain injury team (Team D) in NHS Lanarkshire, Scotland took part. Teams comprised a total of 48 multi-disciplinary staff members. See Supplementary File S3 for a descriptor of each participating team and usual goal setting practice pre-training delivery.

#### 2.2.2 Delivery of the online G-AP training resource

The G-AP website link was emailed to all rehabilitation staff who were asked to dedicate three hours to complete the training within a four-week period. Staff were then invited to take part in webinar 1 then webinar 2, one week later. Webinars were led by LS supported by one other project group member (SB, II or LG). Webinars were recorded and made available to staff for review or catch up. The G-AP training website remained available to staff throughout the study period.

#### 2.2.3 Evaluation design and procedure

A mixed methods evaluation using a convergent design (26, pg. 52) was conducted to evaluate the G-AP training resource and its impact on early (one month post training) G-AP implementation. Informed by the convergent design, qualitative and quantitative data were collected then combined to provide different insights about staff opinions of the G-AP training and their experiences of implementing G-AP in practice. Combining these data sets provided a more complete training evaluation. All rehabilitation staff from participating teams were eligible to take part. The criteria for describing and evaluating training interventions in healthcare professions—CRe-DEPTH (27) were used to inform the conduct and reporting of this study (see Supplementary File S4).

#### 2.2.4 Data collection

##### 2.2.4.1 G-AP training questionnaire

Building on a previous questionnaire (19), a G-AP training questionnaire was developed and emailed to staff within one week of completing the training website and webinars (see Supplementary File S5).

##### 2.2.4.2 Staff focus group

A one hour focus group was conducted following the training period to explore staff opinions and experiences of the G-AP online training and webinars and the extent to which they had prepared them to implement G-AP in practice. Staff were purposively sampled to ensure all professional groups and levels of experience across the three teams were represented.

#### 2.2.5 Data analysis

Informed by our mixed methods convergent design (26), each data set was analysed separately then combined to address the research questions. G-AP training questionnaire data were analysed using descriptive statistics; open ended responses were categorised and described. Focus group data were analysed using a five stage Framework approach (28, 29) (see Supplementary File S6). A conceptual overview was developed [KE, LS] to illustrate the combined data set.

#### 2.2.6 Approvals

Ethical approval was obtained from the Glasgow Caledonian University Nursing Department Research Ethics Committee (ref: HLS/NCH/21/010). No NHS ethics approval was required as no patient participants were included in this study.

## 3 Results

### 3.1 Participants

Eighty five percent of staff (41/48) responded to the G-AP training questionnaire, of which 8/48 (17%) participated in the focus group (see Figure 2). All 41 respondents completed the online training and 40 attended both webinars.

### 3.2 Staff opinions and experiences of the G-AP training resource

**Figure 3:**
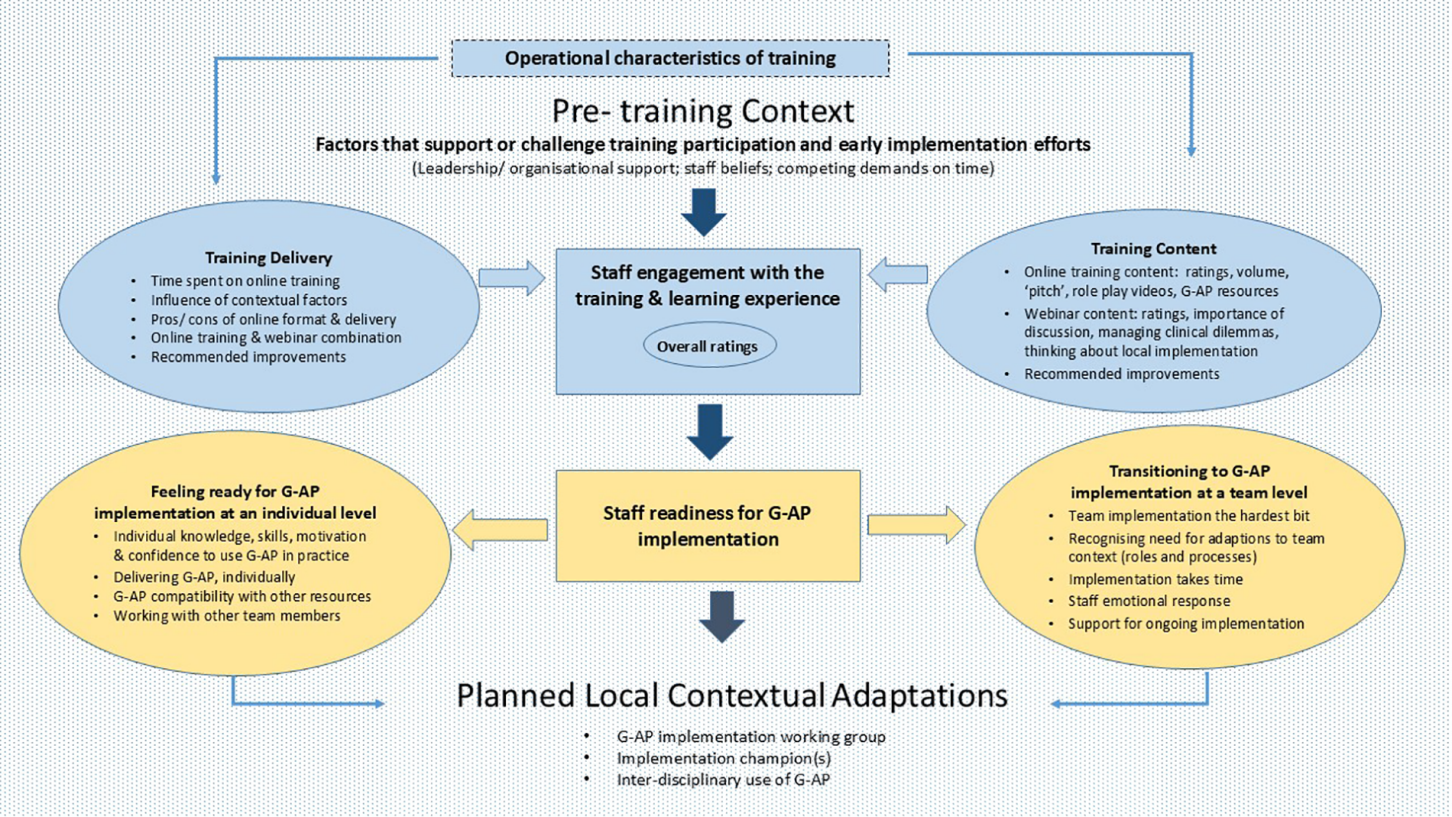
Conceptual thematic overview incorporating contextual factors.

Quantitative and qualitative data are combined and reported under the main themes of *staff engagement with the training, learning experience* and *readiness for G-AP implementation*. Illustrative quotes for themes and related sub-themes are referenced within the text (e.g., Quote 1) and presented in Table 2. A conceptual thematic overview incorporating contextual factors, is presented in Figure 3.

**Table 2:** Themes, sub-themes and illustrative quotes.

Theme	Subtheme	Illustrative quotes
Staff engagement with the training	Contextual factors influencing staff engagement	Q1 “*Busy caseload and lots of competing development projects on the go within the team at the moment.*” (*QR 4; Question 5) Q2 “*Obviously you've got the external issues you won't always have like the Covid aspect, the amount of staff that are maybe off and the extra pressures that are there that hopefully in the future you're not going to have to deal with*.” [Focus Group; P1] Q3 “*We are always fire-fighting with regards to clinical time and we are always really busy, but as long as we know it's* [G-AP training] *so important, related to our work and it's going to make things better in the long run, then it's about managing it. So, I was happy to fit it in and work round as best I could*.” [Focus Group; P8] Q4 “[My] *manager was supportive to schedule a half day working from home to allow training without distraction which was helpful.*” (Response 12, Question 5). Q5 “*What was helpful was having plenty of notice about the training, therefore I could book the training into my diary a good few weeks in advance*.” (QR 16, Question 5).
	Delivery of the online G-AP training and webinars: pros and cons	Q6 “*I thought it* [the G-AP training website] *flowed really well. It was easy to navigate. You could dip in and out of the sections—they were self-explanatory*.” [Focus Group, P6] Q7 “*Because it was online, it didn't take you all day. If you were going to a course, by the time you get there and by the time you get back. I thought that was a bit easier as well. It wasn't as time consuming*.” [Focus Group; P2] Q8 “*It* [the website training] *has pros and cons. I guess it depends on your own learning style as well, doesnt it? What you prefer to do*.” [Focus Group; P7]. Q9 “*Getting peoples opinions is a bit harder when you're online than when you are face-to-face, maybe people open up a bit more* [face-to-face].” [Focus group; P4] Q10 “*It was helpful to have that time to go through it* [the G-AP training website] *all yourself and do all the reading and have that understanding of what it* [G-AP] *is and then go into the discussion groups* [webinars].” [Focus Group; P7] Q11 “*It would be helpful to have trialled G-AP with a few patients prior to webinar* [B], *then we could bring to discussions what worked well/what didn't more, as it's the practical side of implementing it that's the challe*nge.” (QR 9, Question 33).
Staff learning experience	G-AP website content	Q12 “*I think the content of it [the G-AP training website] was really comprehensive. It covered everything that you wanted to hear about. But it was quite a lot to tackle in one go so you do need to split it up*.” [Focus Group; P3] Q13 “*For staff experienced in goal setting, I did not feel this* [videos] *added to my learning; several videos also increased time spent completing training*.” (QR 8, Question 12). Q14 “*I think the videos were very good and very helpful. I think practically you can understand, well I can, a lot easier if you can see it being played out rather than just reading lots of scrolls of writing*.” (Focus Group; P4)
	G-AP webinar content	Q15 “*It was good to interact with all members of rehabilitation team* [in the webinars]” (QR 3, Question 33) Q16 “[The webinars were] *informal enough to feel safe to ask questions*” (QR 4, Question 38). Q17 “*It was really helpful to have that discussion* [in webinar A] *of what it* [G-AP] *actually looks like in everyday working life and the practicalities around it. The different patients it might work well with, and the patients it might be more challenging with*.” [Focus group, P7] Q18 “*I definitely thought the implementation webinar* [webinar B] *was really useful to gather the information* [about local implementation].” [Focus group, P4] Q19 “*I think the whole point about goal setting with your patients is that it is really steeped in communication, in good communication. When you're not a speech and language therapist, I’ll put my hands up, I find those patients* [with communication difficulties] *really, really challenging. So to have the ability to be able to discuss that* [how to support people with communication difficulties] … *to focus on that specific kind of patient was really helpful for me*.” [Focus group, P4] Q20 “*The patients that are really lacking in insight, that aren't quite there yet in terms of setting effective kind of person-centred goals. So, it* [webinar A] *was getting to some of those more tricky bits that you might come up against*.” [Focus group, P3]
Staff readiness for G-AP implementation	Feeling prepared for G-AP implementation at an individual level	Q21 “*I do think it* [the training] *gave you the skills and confidence to go off and use it individually with a patient. I used it with a patient last week and it felt comfortable using it and it worked really well. I used Access G-AP* [accessible version of the G-AP record] *with an aphasic man and I felt the training prepared me to do that*.” [Focus Group; P7] Q22 “*I think when somethings new it's almost like we need permission. Because there was lots of ‘can we make that suggestion? Is that when we step in?’ We need to know that that's OK to do that, so it* [webinar A] *was useful for that*.” [Focus Group, P6] Q23 “*One of my colleagues* [and I], *were working jointly with a patient who has communication difficulties, and today we're going to use the G-AP toolkit. It's actually encouraged us to go back and look at some of the other resources. For this patient I think this* [Talking Mats] *will be a really good tool to use in conjunction with G-AP*.” [Focus Group; P6]
	Transitioning to G-AP implementation at a team level	Q24 “*There is nobody that is not on-board* [with G-AP implementation].” [Focus Group; P8] Q25 “*The implementation is the hard bit. That's the bit that has us scratching our heads about how we are going to make this work logistically, how open people [staff] are to change, how ready are they* [the teams] *to adopt this kind of practice. We all have the knowledge and the understanding and it's what we do with that now.*” [Focus Group; P6] Q26 “*I started using it* [G-AP] *with my own patients and liaising colleagues who have had the same patients, so there's been a bit of multi-disciplinary use as well*.” [Focus Group; P4] Q27 “*I guess the challenge is using it* [G-AP] *alongside other professionals … so physio, OT, stroke nurses and using it not just for the speech therapy goals but as a joint goal setting resource. Just as everyone was saying, it needs more conversations of how that would look*.” [Focus Group; P7]. Q28 “*Our kind of end point from the implementation one* [webinar B] *was ‘right, we need to set up a G-AP implementation working group within our team to figure out how to get through all these things .. we're just setting up our implementation team at the minute*.” [Focus Group; P3] Q29 “*We were talking about an implementation champion and having someone who's got that time to invest. I think that's going to be our main challenge moving forward. We actually really want to use it* [G-AP] *and we want to invest a time but whether that's realistic, I don't know*.” [Focus Group; P7]

*QR, questionnaire response.

#### 3.2.1 Overall ratings

**Figure 4:**
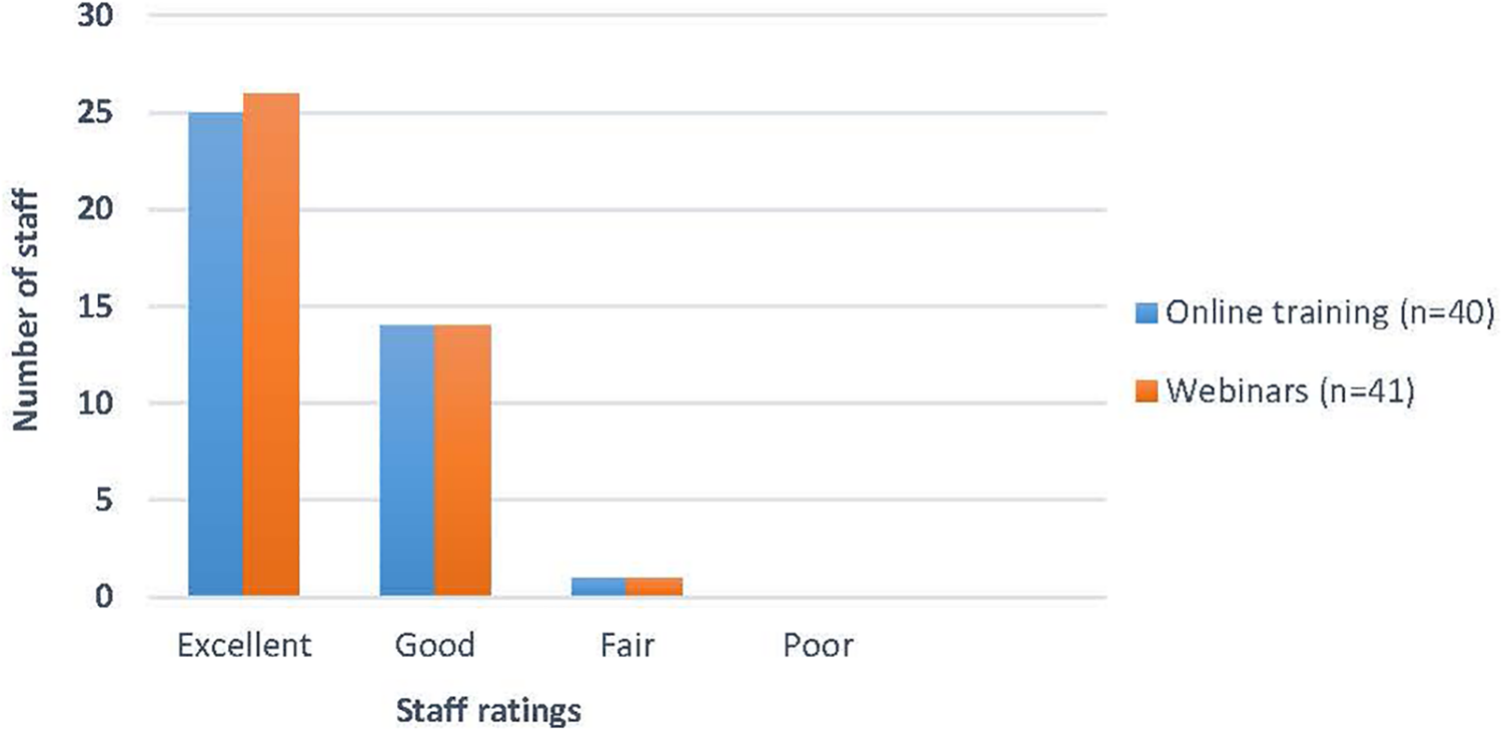
Overall ratings of G-AP training resource.

The vast majority of staff rated the G-AP online training and webinars as either excellent or good (see Figure 4) and reported that the content of the online training (*n* = 36/41; 87%) and webinars (*n* = 31/40; 78%) was ‘very relevant’ to their work with patients. Ninety percent of staff (37/41) agreed that the combination of G-AP website and webinars offered the best learning experience.

#### 3.2.2 Staff engagement with the training

##### 3.2.2.1 Time spent on G-AP training website

**Figure 5:**
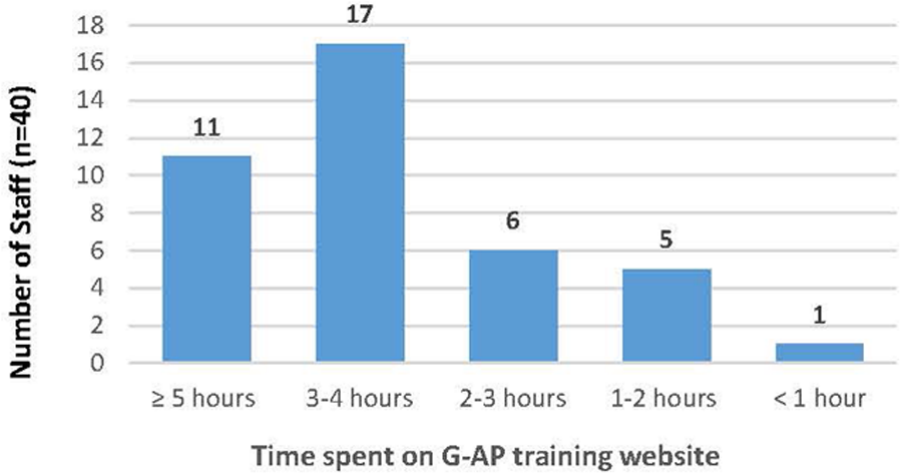
Time spent on G-AP training website.

The majority of staff (28/40; 70%) spent the recommended three hours or more engaging with the G-AP training website (see Figure 5).

##### 3.2.2.2 Contextual factors influencing staff engagement

Sixty-two percent of staff (25/40) reported that setting time aside to complete the online training could be difficult. Local contextual factors such as competing demands, caseload commitments, part-time hours and staffing issues challenged staff engagement (Quote 1). Whilst perceived as a temporary barrier, Covid-19 created an additional challenge to training engagement (Quote 2). Staff beliefs that the training was relevant to their work and would lead to efficiencies in the longer term motivated training engagement (Quote 3). Supportive managers and advanced notice of training were highly valued (Quote 4–5).

##### 3.2.2.3 Pros and cons of online format and delivery of the online G-AP training and webinars

The vast majority of staff reported the G-AP training website was either extremely easy (21/40; 52%) or somewhat easy (*n* = 18/40; 45%) to navigate through (Quote 6) and supported efficient use of time (Quote 7). However, staff acknowledged online training was not everyone's preferred option (Quote 8). The vast majority of staff agreed the webinars were well delivered (40/41; 97%), but 45% (18/41) agreed face to face webinars would have been preferable (Quote 9). Most staff reported that having two webinars of two hour duration was about right (*n* = 35/41; 86%; *n* = 34/41; 83% respectively). Staff liked the combination of the G-AP website and webinars; the majority (*n* = 35/40; 88%) reporting the former was good preparation for the later (Quote 10). However, staff recommended more time between webinar A and B would have supported informed discussions about local G-AP implementation (Quote 11).

#### 3.2.3 Staff learning experience

##### 3.2.3.1 G-AP website content

All sections of the G-AP training website were rated highly (see Table 3). There was consensus that the content was comprehensive, largely pitched at the right level and the amount of information was about right (Quote 12).

**Table 3:** Staff ratings of G-AP training website.

Training section	Excellent *n* (%)	Good *n* (%)	Fair *n* (%)	Poor *n* (%)	N/A *n* (%)	Total responses *n*/41 (%)
About G-AP	22 (55)	17 (43)	1 (2)	0 (0)	0 (0)	40 (98)
G-AP training	20 (50)	18 (45)	1 (2)	0 (0)	1 (2)	40 (98)
Role play videos	23 (58)	12 (30)	3 (8)	1 (2)	1 (2)	40 (98)
Rights, barriers and ramps	23 (58)	15 (37)	0 (0)	0 (0)	2 (5)	40 (98)
Implementation	15 (38)	23 (59)	1 (3)	0 (0)	0 (0)	39 (95)

N/A, not accessed.

Although not everyone agreed the role-play videos provided added value (Quote 13), most staff reported they positively influenced their learning and practice by providing a different training format and illustrating use of G-AP in practice scenarios (Quote 14). Downloadable versions of the G-AP record (including the accessible version—Access G-AP) were rated as ‘very useful’ by 88% (*n* = 35/40) of staff.

##### 3.2.3.2 G-AP webinar content

Staff reported that the webinars were interactive and resulted in a positive learning experience (see Table 4; Quotes 15–16).

**Table 4:** Staff opinions about G-AP webinars.

	Strongly agree *n* (%)	Agree *n* (%)	Disagree *n* (%)	Strongly disagree *n* (%)	Total responses *n*/41 (%)
Webinar discussions supported my learning	25 (62)	14 (35)	1 (2)	0 (0)	40 (98)
I was able to ask questions in the webinars	31 (77)	9 (22)	0 (0)	0 (0)	40 (98)
I found the webinars enjoyable	27 (67)	11 (27)	2 (5)	0 (0)	40 (98)
The webinars were interactive	25 (61)	15 (38)	0 (0)	0 (0)	40 (98)

Webinars supported important discussions that enhanced staff knowledge and confidence to deliver G-AP to individual patients (webinar A) and implement G-AP locally (webinar B) (Quotes 17–18). Staff appreciated opportunities to discuss common clinical dilemmas that impede person-centred practice, for example, supporting patients with communication difficulties and those who may lack insight (Quotes 19–20).

#### 3.2.4 Staff readiness for G-AP implementation

Two main sub-themes captured the extent to which the G-AP training resource supported early G-AP implementation—*Feeling prepared for G-AP implementation at an individual level* and *Transitioning to G-AP implementation at a team level*. Staff felt prepared to initiate G-AP individually. However, implementing G-AP at the team level was perceived to be more challenging and likely to take time for teams to make adaptations to staff roles to action the implementation process and strategies.

##### 3.2.4.1 G-AP implementation at an individual level

**Figure 6:**
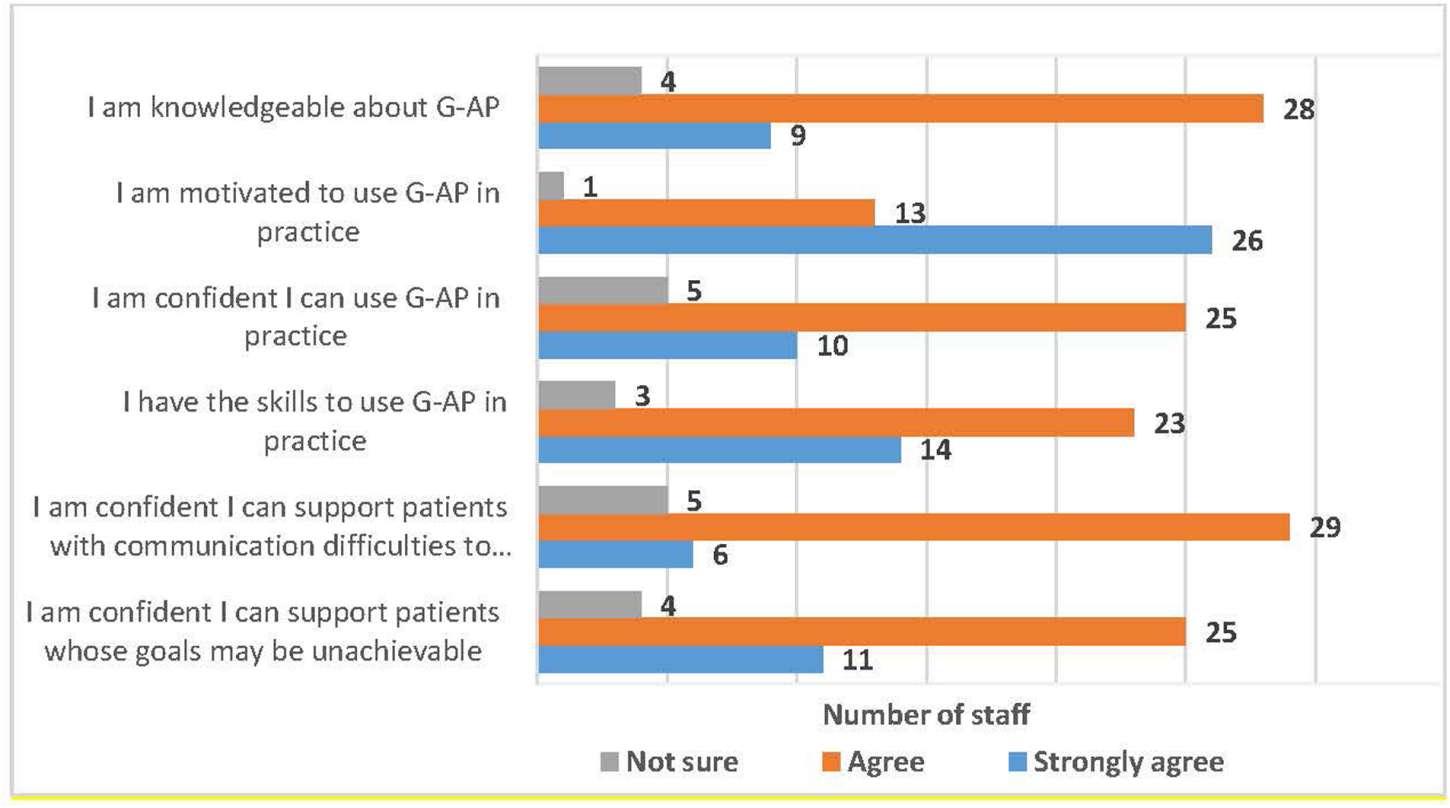
Staff self-ratings of preparedness to used G-AP in practice.

Following training, the vast majority of staff agreed they had the knowledge, motivation, skills and confidence to use G-AP in practice, including supporting patients with communication difficulties and those with potentially unachievable goals (see Figure 6).

Almost all agreed (39/40; 98%) that webinar A supported helpful discussions about use of G-AP with individual patients and had given them the ‘go-ahead’ to try G-AP out in practice. Staff provided examples of implementing G-AP with individual patients, including to those with communication difficulties (Quotes 21–22). G-AP was also used in joint sessions with other staff and viewed as compatible with existing rehabilitation resources (Quote 23).

##### 3.2.4.2 Transitioning to G-AP implementation at a team level

Whilst the vast majority of staff (38/40; 95%) agreed they felt prepared for G-AP implementation, this did not automatically translate into enacting implementation at a team level (Quotes 24–25). Staff recognised that this would require interdisciplinary team work which would take more time to embed (Quotes 26–27). Whilst not yet actioned, staff had taken on board suggested implementation strategies, including setting up a local implementation group and identifying G-AP champions, however having the time to commit to these implementation efforts was a concern (Quotes 28–29).

## 4 Discussion

A fully online G-AP training resource, incorporating a training website and two interactive webinars, has been developed, described and evaluated. The delivery and content of the G-AP training resource were rated highly. The majority of staff felt confident and prepared to deliver G-AP at an individual level and shared examples of practice change. Transitioning to G-AP implementation at a team level was at a preliminary stage and perceived as more challenging and requiring more time. An ongoing interplay existed between local contextual factors and staff engagement with the training, their learning experience and local implementation efforts.

### 4.1 Implementation strategies and temporal considerations

Informed by Expert Recommendations for Implementing Change (25) we used a range of implementation strategies to build a multi-component training resource to support G-AP implementation. Our findings suggest that within one month of training completion, some strategies had yielded positive results. Through our clinical academic partnership, we developed a G-AP training resource of high relevance to clinical practice. Positive staff opinions and experiences of the G-AP training and high ratings of G-AP related skills, confidence and motivation suggests our range of educational materials, resources and delivery methods were highly beneficial to multi-disciplinary staff. However, other implementation strategies needed more time to exert their influence. Whilst local G-AP implementation groups (led by G-AP champions) were planned, more time was needed to convene these groups. Furthermore, G-AP implementation at a team level required some reconfiguration of staff roles and team processes that would take more time to organise. The importance of understanding implementation as a staged process that evolves over time, rather than a one off event, has been highlighted (30, 31). The Stages of Implementation Completion tool (32) tracks implementation across three main stages: *pre-implementation* (engagement and planning), *implementation* (staff training, programme start up, monitoring) and *sustainment* (establishing competency, embedding practice). This study was situated within the pre-implementation and early implementation stage of the process. Evaluation at this stage is critical to pro-actively identify problems or challenges to optimise the chances of later implementation success (32). We are pleased this early evaluation was positive. How G-AP training supports ongoing G-AP implementation and sustainment will be reported in a follow up study.

### 4.2 Training—context interplay

Rehabilitation is a complex process involving delivery of multi-component interventions (23) many of which will rely on staff training as the primary implementation strategy to support their delivery (22). Our results highlighted the influence of local team contexts on staff engagement with the training, their learning experience and subsequent implementation efforts. Evaluations of health care staff training that focus solely on “does it work” are over simplistic and do not take account of this complex training-context interplay (30; pg14). Staff work patterns, beliefs about training relevance (33), high workloads, understaffing and complex workflows (34) are reported contextual factors that have diminished or negated the impact of health care staff training on learning and practice change. Whilst staff training can enhance the requisite knowledge, skills, confidence and motivation required to support practice change and implementation efforts, the training—context interaction will inevitably influence implementation success or failure.

### 4.3 Strengths and limitations

The NHS spends over £4 billion a year on training (35). Reporting the development and evaluation of rehabilitation staff training to support implementation of evidence-based practice is essential to ensure this money is well spent. However, details of training provided to support delivery of rehabilitation interventions is often absent or unclear (23), thus compromising the integrity of the evaluation. Our research has resulted in a G-AP training resource that has been evaluated and can be fully described for both clinical and research purposes. Furthermore, our co-production approach has supported development of a resource that is methodologically sound, clinically relevant and cognisant of patient and carer priorities.

However, this evaluation was conducted in one NHS Scotland board. A large-scale implementation evaluation with more participating NHS sites across the UK is necessary to enhance the generalisability of our findings. The short evaluation time frame and absence of patient data also limit in the extent to which we can report on longer term G-AP implementation and impacts (if any) on patient experiences of care. These limitations are being addressed in our follow up study.

## 5 Conclusions

The co-developed G-AP training resource, incorporating a training website (freely available) and two interactive webinars, has been well received by staff and shows promise in supporting person-centred goal setting practice in community neuro-rehabilitation settings. Implementation was facilitated by training materials that were relevant to clinical practice with clear benefits reflected in improvements to staff skills, confidence and motivation. It is important to recognise that implementation is a staged process that unfolds over time and strategies such as local implementation groups and team reconfiguration take time. Our findings also demonstrate the ongoing interplay between local contextual factors and staff engagement with training, their learning experience and local implementation efforts. Both the temporal nature of training effects and training—context interaction should be factored in when designing rehabilitation training evaluations.

## Data Availability

The raw data supporting the conclusions of this article will be made available by the authors, without undue reservation.
