# Supplementary material for: Development and evaluation of Goal setting and Action Planning (G-AP) training to support person-centred rehabilitation practice
Source: Front Rehabil Sci. 2025 Mar 31;6:1505188. doi: 10.3389/fresc.2025.1505188 (PMC11994713; doi:10.3389/fresc.2025.1505188)
Supplement: Supplementary file 1 [file Datasheet1.pdf]

G-AP training questionnaire FINAL

You have recently been introduced to the **G-AP online training** and **G-AP webinars**. We need your help to **evaluate** the training. Importantly, we would like to identify areas for improvement before the final version of the training and webinars is developed.

We would be **very grateful** if you could complete this G-AP training questionnaire. Please read each question carefully. Some questions are specific to the G-AP online training and others to the G-AP webinars. **Please be honest with your answers** - we know the training and webinars were not perfect and we need your help to make them better!

Please set aside about **10 minutes** to complete the questionnaire. You can complete it on your computer or phone, but it needs to be done in one go as it won't save your responses :) Please complete by **Wednesday 20th April**.

Thanks for your help!

* Required

Questionnaire Consent

Please read the following statements carefully:
1. I have read and understood the study information sheet
2. I have had the chance to talk about the study and ask any questions
3. I understand that it is my choice to take part in the study
4. I understand that I can withdraw from the study at any time without giving a reason and that it is my choice whether the information I have given up until that point is used or not
5. By selecting yes, I give my consent and agree to take part in the questionnaire

1. Do you give your consent to take part in the questionnaire? *

◯ YES

◯ NO

Questions about the online G-AP training

2. Approximately **how much time** did you spend on the G-AP online training in total? *

- 5 hours or more
- 3-4 hours
- 2-3 hours
- 1-2 hours
- Less than 1 hour
- I did not complete the G-AP online training

3. How easy/ difficult was it to **navigate** your way thorough the G-AP online training website? *

- Extremely easy
- Somewhat easy
- Somewhat difficult
- Extremely difficult

4. Was it difficult to **set time aside** to complete the G-AP online training? *

- Yes
- No
- To some extent

5. **Please list any reasons why** it was difficult to set time aside?

6. Would you say the **amount of information** on the G-AP training resource was ... *

- Too much
- About right
- Too little

7. How would you rate the **About G-AP section** of the online training (included content about Why use G-AP; G-AP development & evidence; G-AP theory)

- Excellent
- Good
- Fair
- Poor
- I did not access this section of the training

8. **Please list any improvement(s)** we could make to the About G-AP section of the training

9. How would you rate the **G-AP training section** of the online training (included content about each G-AP stage and Frequently Asked Questions)?

- ◯ Excellent
- ◯ Good
- ◯ Fair
- ◯ Poor
- ◯ I did not access this section of the training

10. **Please list any improvement(s)** we could make to the G-AP training section

[ ]

11. How would you rate the online training **role play videos** (Alice and Tom) as a learning tool? (please tick one response).

- ◯ Excellent
- ◯ Good
- ◯ Fair
- ◯ Poor
- ◯ I did not access this section of the training

12. Please list any improvement(s) we could make to the **role play videos** to enhance your learning

[ ]

13. How would you rate the **Rights, Barriers and Ramps section** of the online training (included content about supporting people with cognitive and communication difficulties)?

- Excellent
- Good
- Fair
- Poor
- I did not access this section of the training

14. **Please list any improvement(s)** we could make to the Rights, Barriers and Ramps section of the training

15. How would you rate the **Implementation section** of the online training (included content about implementing G-AP in your setting)?

- Excellent
- Good
- Fair
- Poor
- I did not access this section of the training

16. **Please list any improvement(s)** you think we could make to the Implementation section

17. How useful was it to have access to the following **additional resources?**

	Very Useful	Somewhat Useful	Not Useful	I did not access these resources
Downloadable versions of the **G-AP record** and **Access G-AP**	◯	◯	◯	◯
G-AP power point **conferen ce presentations**	◯	◯	◯	◯
**Talking Mats** videos	◯	◯	◯	◯
The **Chest Heart and Stroke videos** a bout aphasia and dysarthria	◯	◯	◯	◯
**Access G-AP case study** (Peter)	◯	◯	◯	◯

18. Were there any **topics missing** from the G-AP online training that should have been included? *

◯ Yes

◯ No

◯ Not Sure

19. If yes, can you please list other training topic(s) that should have been included ...

20. **How relevant was the content** of the online G-AP training to your work with patients? *

- ◯ Very relevant
- ◯ Somewhat relevant
- ◯ Somewhat irrelevant
- ◯ Very irrelevant

21. **Was the G-AP online training good preparation for the Webinars?**

- ◯ Yes
- ◯ To some extent
- ◯ No

22. How likely are you to **use the G-AP online training resource again**? *

- ◯ Very likely
- ◯ Somewhat likely
- ◯ Somewhat unlikely
- ◯ Very unlikely
- ◯ Not sure

23. Would you **recommend** the G-AP online training to other rehabilitation staff? *

- Yes
- No
- Not sure

24. Would you **recommend** the G-AP online training to students?

- Yes
- No
- Not sure

25. How would you **rate** the G-AP online training overall?

- Excellent
- Good
- Fair
- Poor
- Very Poor

26. How would you **rate** the G-AP online training overall?

- ◯ Excellent
- ◯ Good
- ◯ Fair
- ◯ Poor
- ◯ Very Poor

Questions about the G-AP webinars

27. Please tell us **how many G-AP webinars** you participated in (either in real time or by catching up via recording): *

- One
- Two
- None

28. If you were **unable to take part** in one or more of the webinars can you briefly explain why?

29. Having **2 webinars (Webinar A and Webinar B)** was... *

- Too much
- About right
- Too little

30. The **length of the webinars** (2 hours) was... *

- Too long
- About right
- Too short

31. To what extent do you agree or disagree with the following statements about the **delivery of the G-AP training webinars?**

	Strongly agree	Agree	Disagree	Strongly disagree
The G-AP training webinars were **well delivered**	◯	◯	◯	◯
Webinar **discussions** supported my learning	◯	◯	◯	◯
I was able to **ask questions** in the webinars	◯	◯	◯	◯
I found the webinars **enjoyable**	◯	◯	◯	◯
There were no **distractions** which disrupted my learning	◯	◯	◯	◯
**Microsoft TEAMS** was a good platform to deliver the webinars on	◯	◯	◯	◯
**Face to face** training would have been preferable	◯	◯	◯	◯
The webinars were **interactive**	◯	◯	◯	◯

32. To what extent do you agree or disagree with the following statements about the **content of the G-AP training webinars ...**

	Strongly agree	Agree	Disagree	Strongly disagree
Webinar content was **relevant** to my work with patients	◯	◯	◯	◯
Webinar content helped me **prepare for G-AP implementation**	◯	◯	◯	◯
The webinars **supported discussion** about how to use G-AP with different patients	◯	◯	◯	◯
The webinars **supported my learning about G-AP**	◯	◯	◯	◯

33. Do you have any comments or suggestions about the **content** of the webinars?

34. Would you be happy to take part in a **follow up Implementation Webinar** in three months time?

- ◯ Yes
- ◯ Not Sure
- ◯ No

35. How would you rate the **G-AP webinars** overall? *

- ◯ Excellent
- ◯ Good
- ◯ Fair
- ◯ Poor
- ◯ Very Poor

General questions

36. Please state **to what extent you agree or disagree** with the following statements:

	Strongly agree	Agree	Not sure	Disagree	Strongly disagree
I am **knowledgeable** about G-AP	○	○	○	○	○
I am **motivated** to use G-AP in practice	○	○	○	○	○
I am **confident** I can use G-AP in practice	○	○	○	○	○
I have the **skills** to use G-AP in practice	○	○	○	○	○
I am confident I can support patients with **communication difficulties** to use G-AP	○	○	○	○	○
I am confident I can support patients with **cognitive difficulties** to use G-AP	○	○	○	○	○
I am confident I can support patients to set **person centred goals**	○	○	○	○	○
I am confident I can support patients whose goals **may not be achievable**	○	○	○	○	○

37. In your opinion, which of the following training options would be the **best learning experience** for you?

- ◯ G-AP online training only
- ◯ G-AP webinars only
- ◯ G-AP online training AND G-AP webinars

38. **Is there anything else you would like to tell us** about the online G-AP training or the G-AP webinars? Please let us know in the box below ...

Questions about you

39. Which **professional group** do you belong to? *

- ◯ Physiotherapy
- ◯ Speech and language therapy
- ◯ Occupational therapy
- ◯ Assistant practitioner (any professional group)
- ◯ Nurse
- ◯ Psychologist
- ◯ Student nurse or allied health professional
- ◯ Other

40. What is your **Band** or **Grade**?

41. Which **team** do you belong to? *

- North Stroke and Neuro Rehab Team
- South Stroke and Neuro Rehab Team
- Brain Injury Team
- Stroke MCN team
- Other

Thank you for taking the time to complete this post G-AP training survey!

This content is neither created nor endorsed by Microsoft. The data you submit will be sent to the form owner.

Microsoft Forms
