# Supplementary material for: Development and evaluation of Goal setting and Action Planning (G-AP) training to support person-centred rehabilitation practice
Source: Front Rehabil Sci. 2025 Mar 31;6:1505188. doi: 10.3389/fresc.2025.1505188 (PMC11994713; doi:10.3389/fresc.2025.1505188)
Supplement: Supplementary file 2 [file Table1.docx]

|  | **Team members** | **Expertise** | **Contribution to G-AP training resource** |
| --- | --- | --- | --- |
| **Clinical-academic project team**  **(n=5)** | Clinical-academic occupational therapist (LS)  Clinical-academic speech and language therapist (SB)  Practicing speech and language therapists (EC,LG)  Practicing Physiotherapist (II) | Expertise in the G-AP research and development; clinical expertise delivering goal setting to people with neurological conditions  Clinical expertise delivering goal setting to people with neurological conditions | Developed content of the new G-AP training resource, including video material and webinars |
| **Advisory group**  **(n=8)** | People with neurological conditions, including one person with a communication difficulty (n=3); Carers (n=2)  Rehabilitation staff members (n=3) | Lived experience of neurological conditions, rehabilitation & setting / pursuing personal goals  Clinical expertise delivering goal setting to people with neurological conditions | Reviewed and provided feedback on the developing training content |
