# Supplementary material for: Development and evaluation of Goal setting and Action Planning (G-AP) training to support person-centred rehabilitation practice
Source: Front Rehabil Sci. 2025 Mar 31;6:1505188. doi: 10.3389/fresc.2025.1505188 (PMC11994713; doi:10.3389/fresc.2025.1505188)
Supplement: Supplementary file 3 [file Table2.docx]

| **Additional G-AP website content** | **Training objective** | **What informed content development?** |
| --- | --- | --- |
| ***Rights, Barriers and Ramps section:***  Included ***information*** about (i) everyone’s RIGHT to rehabilitation, (ii) the communication and/ or cognitive difficulties that act as a BARRIERS to patient involvement in the goal setting process and (iii) targeted strategies and resources (RAMPS) that can support optimal patient involvement.  ***‘Talking heads’ video clips*** included to illustrate communication and cognitive RAMPS.  ***Illustrated Case study example*** of using Access G-AP with a person with aphasia. | Staff will support all patients, including those with communication and/ or cognitive difficulties, to be optimally involved in the G-AP process | *Rehabilitation policy:*   - Rehabilitation is a human right and should be available to all who need it (including those with communication and/ or cognitive difficulties) [1]   *Research evidence:*   - Cognitive and/ or communication difficulties are consistently reported as barriers to patient engagement in the goal setting process [2,3,4] - Staff lack confidence setting goals with stroke survivors with aphasia; staff training to support accessible goal setting with stroke survivors with aphasia is an unmet need; greater staff awareness of communication support tools is required [5] - Access G-AP has been developed to support people with aphasia through the G-AP process [6] - Staff knowledge, skills and confidence is enhanced through information provision and illustrating/ describing use of communication support strategies and resources (RAMPS) and Access G-AP in clinical scenarios [7-8]   *Clinical experience:*   - Collective experience and knowledge of the project team informed identification of strategies and resources (RAMPS) to support optimum patient involvement at each stage of the G-AP process. - ‘Talking heads’ video clips were delivered by practicing clinicians and informed by their clinical expertise and experience |
| ***Implementation section:***  ***Implementation*** ***information*** and ***resources*** to support planning for local G-AP implementation, including (i) assessing the ‘fit’ between current goal setting practice and the G-AP process, (ii) useful questions to consider when planning for G-AP implementation, (iii) prompting the use of G-AP in day to day practice, (iv) setting up a local G-AP implementation group and (v) tailoring G-AP to individual patients | Staff will work together to plan for and implement G-AP in their local setting | *Research evidence:*   - Standalone education interventions are not effective in translating evidence into practice; multi-component interventions including education, implementation facilitation and local tailoring are effective [9] - Planning for implementation as a ‘critical step’ to implementation success [10] - G-AP training should include content about planning for local G-AP implementation, including identifying G-AP ‘champions’ within the team and developing a local implementation plan [11] - Use reminders to prompt habits to support G-AP implementation [7] |
| ***Role play video material:***  Case study based role play videos illustrate different approaches to delivering G-AP in practice. ‘Reflective questions’ attached to each video encourage reflection | Staff will reflect on their own practice and seek to maintain a person centred approach to G-AP delivery | *Research evidence*:   - Rehabilitation staff report preference for training that consists of workshops and videos [5] - Make training dynamic by varying the information delivery methods [12] - Role play and modelling of target behaviours to improve staff knowledge, skills, habits and confidence [7,8]   *Advisory group feedback:*   - Role play videos should not illustrate a ‘right’ or ‘wrong’ approach to G-AP conversations; instead they should prompt staff to reflect on and discuss what worked well or not so well in each role play scenario. |
| ***Resources section:***  Editable version of the G-AP record and Access G-AP available to review/ download.  Links to recorded G-AP presentations available. | Staff will have G-AP resources available to support their local practice and implementation efforts. G-AP resources will be modifiable for local use. | *Research evidence*:   - Interventions which can be adapted and tailored to meet local need are more likely to be implemented [12]   *Advisory group feedback:*   - Some staff may prefer to watch recorded G-AP presentations rather than written content in the online training content to support their learning. |
| ***Additional subsections:***  ‘Hope and positivity’ section highlights the importance of maintaining a hopeful and positive approach throughout the G-AP process. | Staff will understand the importance maintaining a hopeful and positive approach in their interactions with patients. | *Advisory group feedback:*   - Based on their personal experiences, patients and carer advisors highlighted the importance of staff maintaining a hopeful and positive approach throughout the G-AP process. |
| ***Academic papers:***  G-AP and other academic papers were included as optional ‘further reading’ within each section of the training | Staff who desire more in-depth knowledge and understanding of G-AP and its delivery in practice will have easy access to relevant academic papers to inform their practice | *Research evidence:*   - Staff will feel more confident and motivated to implement G-AP if they believe it is evidence based, credible and aligned to their professional standards of practice [7]   *Advisory group feedback:*   - Staff are likely to differ in the amount and depth of information they would like and/ or need to inform their practice. Access to academic papers as ‘optional reading’ would be beneficial, especially to those staff who would like to develop their knowledge further and understand the evidence underpinning the G-AP training content. |
| **Webinar content** | **Training objective** | **What informed content development?** |
| Two webinars developed to enable synchronous remote training incorporating peer learning and support.  **Webinar A:**  Delivering G-AP to individual patients; managing common clinical dilemmas  **Webinar B:** Planning for G-AP implementation in your team; anticipating local barriers and how to overcome them | G-AP training will include an interactive component for questions, discussion and peer learning.  Staff will be confident to manage/ navigate common clinical dilemmas during the G-AP process.  The multi-disciplinary team will work together to implement G-AP at an individual patient and team level. | *Research evidence*   - Education delivered alongside multi-disciplinary colleagues can support discussion of the practicalities of local implementation [13] - Multi-component training interventions that included an element of site-specific tailoring (e.g. workshops to examine local barriers and ways to overcome them) are recommended [9] - Staff will feel motivated to implement G-AP if they are confidence that can manage common clinical dilemmas and are knowledgeable of implementation strategies and how they might be helpful [7]   *Clinical experience/ Advisory group feedback*   - Clinicians report common goal setting dilemmas e.g. should goals be SMART? Do patients need to come up with their own goals? What if a patient’s goal is unrealistic? These common dilemmas should be addressed within the G-AP training. |

**References**

[1] Skempes D, Stucki G, Bickenbach J. Health-related rehabilitation and human rights: analyzing states' obligations under the United Nations Convention on the Rights of Persons with Disabilities. Arch Phys Med Rehabil 2015; 96(1):163-173.

[2] Plant S,E., Tyson S,F., Kirk S, Parsons J. What are the barriers and facilitators to goal-setting during rehabilitation for stroke and other acquired brain injuries? A systematic review and meta-synthesis. Clin Rehabil 2016; 30(9):921-930.

[3] Rosewilliam S, Roskell C, Pandyan A. A systematic review and synthesis of the quantitative and qualitative evidence behind patient-centred goal setting in stroke rehabilitation. Clin Rehabil 2011; 25:501-514.

[4] Sugavanam T, Mead G, Donaghy M, van Wijke F. The effects and experiences of goal setting - A systematic review. Disabil Rehabil 2013; 35(5):177.

[5] Brown SE, Scobbie L, Worrall L, Brady MC. A multinational online survey of the goal setting practice of rehabilitation staff with stroke survivors with aphasia. Aphasiology 2023a; 37(3):479-503.

[6] Brown SE, Scobbie L, Worrall L, Mc Menamin R, Brady MC. Access G-AP: development of an accessible goal setting and action planning resource for stroke survivors with aphasia. Disabil Rehabil 2023b; 45(13):2107-2117.

[7] Michie S, Johnston M, Abraham C, Lawton R, Parker D, Walker A, et al. Making psychological theory useful for implementing evidence based practice: a consensus approach. Qual Saf Health Care 2005; 14(1):26-33.

[8] Michie S, Johnston M, Francis J, Hardeman W, Eccles M. From theory to intervention: Mapping theoretically derived behavioural determinants to behaviour change techniques. Applied Psychology: An International Review 2008; 57(4):660-680.

[9] Bird M, Miller T, Connell LA, Eng JJ. Moving stroke rehabilitation evidence into practice: a systematic review of randomized controlled trials. Clin Rehabil 2019; 33(10):1586-1595.

[10] Ross J, Stevenson F, Lau R, Murray E. Factors that influence the implementation of e-health: a systematic review of systematic reviews (an update). Implementation Science 2016;11(146).

[11] Scobbie L, Duncan EAS, Brady MC, Thomson K, Wyke S. Facilitators and “deal breakers”: a mixed methods study investigating implementation of the Goal setting and action planning (G-AP) framework in community rehabilitation teams. BMC Health Services Research 2020; 20(1):791.

[12] Powell BJ, Waltz TJ, Chinman MJ, Damschroder LJ, Smith JL, Matthieu MM, et al. A refined compilation of implementation strategies: results from the Expert Recommendations for Implementing Change (ERIC) project. Implementation Science 2015; 10(2).

[13] Jones F, Bailey N. How can we train stroke practitioners about patient self-management? Description and evaluation of a pathway wide training programme. European Journal for Person Centred Healthcare 2013; 1:246.
