# Supplementary material for: Development and evaluation of Goal setting and Action Planning (G-AP) training to support person-centred rehabilitation practice
Source: Front Rehabil Sci. 2025 Mar 31;6:1505188. doi: 10.3389/fresc.2025.1505188 (PMC11994713; doi:10.3389/fresc.2025.1505188)
Supplement: Supplementary file 4 [file Table3.docx]

| **Participating Teams** | | | | |
| --- | --- | --- | --- | --- |
|  | **Team A** | **Team B** | **Team C** | **Team D** |
| **Patient diagnostic groups** | Community dwelling adults with stroke or neuro condition | Community dwelling adults with stroke or neuro condition | Community dwelling adults with stroke | Community dwelling adults with traumatic brain injury |
| **How long team established** | > 10 years | > 10 years | > 10 years | > 10 years |
| **Number of rehab. staff** | 12 | 10 | 10 | 16 |
| **Team members** | ARP (n=2)  OT (n=4)  Physio (n=5)  SLT (n=1) | ARP (n=1)  OT (n=2)  Physio (n=3)  SLT (n=4) via referral* | Stroke nurse (n=7)  OT (n=2)  Physio (n=1) | ARP (n=3)  OT (n=6),  Physio (n=3)  SLT (n=2)  Psych (n=2) |
| **Bands * represented** | 4-7 | 4-7 | 6-7 | 4-8a |
| **Duration of rehab input** | 1-22 weeks | 5-12 weeks | Open ended | 5-22^+^ weeks |
| **Usual goal setting practice** | | | | |
| **Is goal setting used?** | Yes - with all/ most patients | Yes - with most patients | Team C supports attainment of goals set by Team A & B. Staff attend MDT/ goal discussion meetings in Team A & B on a patient by patient basis | Yes - with all/most patients |
| **Patient involvement in setting goals** | Individual team members set goals with patients | Individual team members set goals with patients |  | Individual team members set goals with patients |
| **Goal setting methods used** | Team/ individual team members developed own method | Team developed own method; SMART goals |  | Team developed own method; SMART goals |
| **MDT meetings** | weekly | weekly |  | weekly |
| **Priority given to goal setting** | high | high |  | high |
| **Neuro:** Neurological; **MDT:** Multi-disciplinary team; **ARP**: Assistant Rehabilitation Practitioner; *** Bands:** Agenda for Change NHS staff Bands - <https://www.healthcareers.nhs.uk/working-health/working-nhs/nhs-pay-and-benefits/agenda-change-pay-rates>; **SMART**: Specific, Measurable, Achievable, Realistic and Timed. | | | | |
