# Supplementary material for: Development and evaluation of Goal setting and Action Planning (G-AP) training to support person-centred rehabilitation practice
Source: Front Rehabil Sci. 2025 Mar 31;6:1505188. doi: 10.3389/fresc.2025.1505188 (PMC11994713; doi:10.3389/fresc.2025.1505188)
Supplement: Supplementary file 5 [file Table4.docx]

| **CRe-DEPTH Criteria** | **Where details can be found within manuscript** |
| --- | --- |
| **1: Description of the aim or objectives of the training** | - Overall aim of the study: Pg. 3; Para 4. - The specific objectives of the training are detailed on the landing page of the G-AP training website: <https://g-apframework.scot/> and Additional File 2. |
| **Item 2: Description of the underlying theoretical framework** | The theoretical framework informing development of the training is described:   - Pg. 4 (Development of the new training content) - Pg. 4 (Use of implementation strategies) - Additional File 2. - Figure 1. (G-AP implementation strategies) |
| **Item 3: Description of the developmental** **process** | Development of the training is described:   - Pg. 4 (Co-production approach; development of the new training content) |
| **Item 4: Description of target population and setting of the training** | Target population of the training and training setting is described:   - Pg. 5 (Participating teams) - Additional File 3 (Participating teams and usual goal setting practice) |
| **Item 5: Description of the educational resources** | - Table 1 (Overview of the online G-AP training resource) - G-AP training website link: <https://g-apframework.scot/> |
| **Item 6: Description of the content of the training** | - As above |
| **Item 7: Description of the format** | Format and delivery of training are described:   - Pg. 5 (Delivery of the online G-AP training resource) - Figure 2 (Study participants and procedure) |
| **Item 8: Description of the didactic methods of training** | - Knowledge required to understand and deliver G-AP in practice was included in the G-AP training website. The training sections are described in Table 1 (Overview of the online G-AP training resource) - Webinars were did not include didactic methods. They focused on group discussion and peer learning. |
| **Item 9: Description of tailoring of the training** | - The G-AP training website could be accessed 24/ 7 over a 4 week period at a time convenient to staff participants. - Participants were invited to take part in G-AP webinars x 2. Webinar recordings were available to anyone not able to attend the webinars. - Pg. 5 (Delivery of online G-AP training resource) |
| **Item 10: Description of the providers of the training** | - Pg. 5 (Delivery of the online G-AP training resource) |
| **Item 11: Description of the measured outcomes** | - Pg. 6 (Data Collection) - G-AP training questionnaire (Additional File 5) |
| **Item 12: Description of the applied assessment method, including validity and reliability** | - Pg. 5 (Evaluation design and procedure) - Pg. 6 (Data Collection) - Pg. 6 (Data Analysis) |

**Reference**

**Van Hecke** A, Duprez V, Pype P, Beeckman D, Verhaeghe S. Criteria for describing and evaluating training interventions in healthcare professions - CRe-DEPTH. Nurse Educ Today. 2020; 84:104254. doi: 10.1016/j.nedt.2019.104254. Epub 2019 Oct 22. PMID: 31689586.
