# Supplementary material for: Development and evaluation of Goal setting and Action Planning (G-AP) training to support person-centred rehabilitation practice
Source: Front Rehabil Sci. 2025 Mar 31;6:1505188. doi: 10.3389/fresc.2025.1505188 (PMC11994713; doi:10.3389/fresc.2025.1505188)
Supplement: Supplementary file 6 [file Table5.docx]

| **Stage** | **Analysis process** |
| --- | --- |
| Stage 1 | The transcript was checked against the audio recording and anonymised [KE] to ensure the accuracy of and established familiarisation with the whole data set. Notes on broad themes and initial impressions were recorded and discussed [KE, LS]. The anonymised transcript was imported into QSR International NVivo 12 qualitative data analysis software to facilitate data management [KE]. |
| Stage 2 | The transcript was read and codes (paraphrases/ labels) applied to data excerpts to identify broad expected (e.g. views about training content) and novel (e.g. interaction between context and training engagement) themes [KE]. The broad thematic framework was reviewed and agreed [KE, LS]. |
| Stage 3 | The agreed thematic framework was applied to the whole transcript [KE] and data within each broad theme were reviewed and coded into sub-themes [KE]. Redundant sub-themes were removed, overlapping themes merged and others re-labelled to better reflect the data contained within them [KE, LS]. The final thematic framework was discussed and approved with the project team [LS, KE, RF, MB]. |
| Stage 4 | Themes were mapped and summarised into an NVIVO framework matrix, links to original data were maintained [KE]. |
| Stage 5 | A thematic conceptual diagram was iteratively developed [KE, LS] and approved [RF, MB] to illustrate how characteristics of the training and contextual factors influenced G-AP implementation at the individual and team level. |
